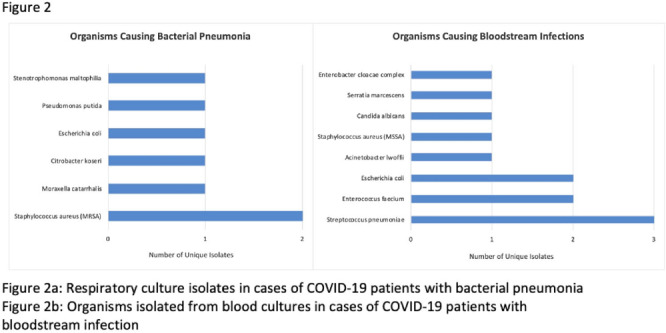# Coinfections in hospitalized COVID-19 patients are associated with high mortality: need for improved diagnostic tools

**DOI:** 10.1017/ash.2022.67

**Published:** 2022-05-16

**Authors:** Sonya Kothadia, Brigid Wilson, Federico Perez, Robert Bonomo

## Abstract

**Background:** Hospitalized patients with COVID-19 often receive antimicrobial therapies due to concerns for bacterial and fungal coinfections. We analyzed patients admitted with COVID-19 to our VA facility to understand antimicrobial use, frequency of coinfections, and outcomes in our population. **Methods:** This retrospective study included veterans who were 18 years or older and hospitalized with COVID-19 from March 10, 2020, to March 9, 2021 at the Louis Stokes VA Medical Center in Cleveland, Ohio. We identified antimicrobials administered and coinfections with bacterial or fungal pathogens. Patients were deemed to have coinfection if there was supporting microbiological data and a consistent clinical course upon review of clinical records. Urinary tract infections were excluded because of difficulty determining infection. Odds ratios (ORs) and 95% confidence intervals (CIs) for 30-day mortality were derived using multivariate logistic regression models that included age, Charlson comorbidity index (CCI), corticosteroid use, and time of infection. **Results:** In our cohort of 312 patients, the median age was 70 years and 97% of the patients were male. The mean CCI was 3.7 (SD, 3.0), and 111 patients (35.6%) had a score ≥5. Oxygen was administered to 250 patients (80.1%), and 20 (6.4%) required mechanical ventilation. Antimicrobials were administered to 164 patients (52.6%) (Fig. 1). Of 20 patients (6.4%) with coinfection, 11 (3.5%) had a bloodstream infection (BSI) and 9 (2.9%) had bacterial pneumonia (Fig. 2). The overall 30-day mortality rate was 12.5% (39 of 312). Among patients with coinfection, the 30-day mortality rate was 45% (9 of 20). Diagnoses of BSI (OR, 6.35; 95% CI, 1.41–26.30) and bacterial pneumonia (OR, 9.34; 95% CI, 2.01–46.34) were associated with increased mortality. Of the data available, 12 (63%) of 19 patients with coinfection had elevated procalcitonin levels (ie, >0.50). At the time of COVID-19 diagnosis, the median absolute lymphocyte count in patients who died was 0.7 K/mm^3^ (95% CI, 0.6–1.12) in comparison to 1 K/mm^3^ (95% CI, 0.7–1.4) in patients who survived at 30 days. **Conclusions:** Our analysis of hospitalized COVID-19 patients with advanced age and underlying comorbid conditions demonstrated that coinfections were infrequent but that they were independently associated with increased mortality. This finding highlights the need for better tools to diagnose the presence or absence of bacterial and fungal coinfection in COVID-19 patients. Our findings also emphasize the need for judicious use of antimicrobials while discerning which patients are at risk of critical illness and mortality.

**Funding:** None

**Disclosures:** None

**Figure:**
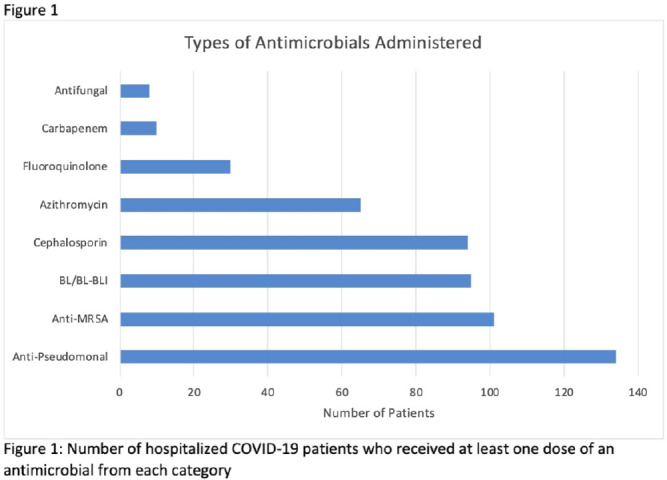

**Figure:**